# Longitudinal study of lung function in pregnant women: Influence of parity and smoking

**DOI:** 10.6061/clinics/2017(10)02

**Published:** 2017-10

**Authors:** Luciana Duzolina Manfré Pastro, Miriam Lemos, Frederico Leon Arrabal Fernandes, Silvia Regina Dias Médici Saldiva, Sandra Elisabete Vieira, Beatriz Mangueira Saraiva Romanholo, Paulo Hilário Nascimento Saldiva, Rossana Pulcineli Vieira Francisco

**Affiliations:** I Departamento de Ginecologia e Obstetricia, Faculdade de Medicina FMUSP, Universidade de Sao Paulo, Sao Paulo, SP, BR; II Departamento de Patologia, Faculdade de Medicina FMUSP, Universidade de Sao Paulo, Sao Paulo, SP, BR; III Laboratorio de Funcao Pulmonar, Disciplina de Pulmonologia, Instituto do Coracao (InCor), Hospital das Clinicas HCFMUSP, Faculdade de Medicina, Universidade de Sao Paulo, Sao Paulo, SP, BR; IV Instituto de Saude, Secretaria de Estado da Saude, Sao Paulo, SP, BR; V Departamento de Pediatria, Faculdade de Medicina FMUSP, Universidade de Sao Paulo, Sao Paulo, SP, BR; VI Departamento de Medicina (LIM 20), Faculdade de Medicina, Universidade de Sao Paulo, Sao Paulo, SP, BR; VII Universidade Cidade de Sao Paulo (UNICID), Sao Paulo, SP, BR

**Keywords:** Spirometry, Pregnancy, Pulmonary Function Test, Parity, Smoking

## Abstract

**OBJECTIVES:**

To evaluate pulmonary function in the first and third trimesters of pregnancy and analyze the influence of parity and smoking on spirometry parameters.

**METHODS:**

This longitudinal prospective study included a cohort of 120 pregnant women. The inclusion criteria were as follows: singleton pregnancy, gestational age less than 13.86 weeks, and no preexisting maternal diseases. The exclusion criteria were as follows: change of address, abortion, and inadequate spirometry testing. ClinicalTrials.gov: NCT02807038.

**RESULTS:**

A decrease in values of forced vital capacity and forced expiratory volume were noted in the first second from the first to third trimester. In the first and third trimesters, multiparous women demonstrated lower absolute forced vital capacity and forced expiratory volume values in the first second compared with nulliparous women (*p*<0.0001 and *p*=0.001, respectively). Multiparous women demonstrated reduced forced expiratory flow in 25% to 75% of the maneuver compared with nulliparous women in the first (*p*=0.005) and third (*p*=0.031) trimesters. The absolute values of forced expiratory flow in 25% to 75%, forced expiratory volume in the first second and predicted peak expiratory flow values in the third trimester were higher in smokers compared with nonsmokers (*p*=0.042, *p*=0.039, *p*=0.024, and *p*=0.021, respectively).

**CONCLUSION:**

There was a significant reduction in forced vital capacity and forced expiratory volume values in the first second during pregnancy. Parity and smoking significantly influence spirometric variables.

## INTRODUCTION

Pregnancy is a period during which the female body undergoes functional and anatomical alterations, including changes in lung function. These findings can be assessed by spirometry, a simple, inexpensive, and effective method 1,2.

There is some controversy regarding the results of spirometry in pregnancy, particularly in terms of tobacco use and parity. Several studies measured lung function during pregnancy and after delivery. Mixed results have been reported for forced vital capacity (FVC), forced expiratory volume in the first second (FEV_1_), and the FEV_1_/FVC ratio with respect to whether they remain the same, increase, or decrease slightly 1,3,4.

The predictors for decreasing lung function during pregnancy remain unknown. Multiparity and smoking status may be associated with decreased lung function.

### Objectives

This study aimed to use spirometry to evaluate changes in pulmonary function in pregnant women between the first (T1) and third trimester (T3) of pregnancy and to evaluate the influence of smoking and parity during this period.

## METHODS

This longitudinal prospective study was conducted from August 2011 to May 2013 at the University of São Paulo Medical School (FMUSP), Brazil. This study was approved by the Ethics Committee for Research Project Analysis at the Hospital of the Faculty of Medicine under number 193/11, and written informed consent was obtained from the subjects. ClinicalTrials.gov: NCT02807038.

### Study population

We included 220 pregnant women who were consecutively recruited from the three primary care units located in the west of the city of São Paulo. The women were part of a larger cohort of 400 patients who were recruited to study the effects of environmental air pollution on pregnancy.

The following inclusion criteria were applied: receipt of prior informed consent, singleton pregnancy, gestational age of less than 13.86 weeks at the first evaluation, and absence of preexisting maternal diseases. The exclusion criteria included withdrawal from the project, abortion, and inadequate spirometry testing performed at T1 and T3.

### Study design

After the confirmation of pregnancy, community health workers made the first contact with the pregnant women. During this first meeting, the research project was explained to the pregnant women, who were invited to participate in the study and provide informed consent. If the women agreed to participate, they received a card with the first assessment date and information about spirometry.

The measurement techniques and spirometry parameters were selected in accordance with the Guidelines for Pulmonary Function Test of the Brazilian Thoracic Association 5. All spirometric values were adjusted according to sex, age, height, and weight at the time of the exam to estimate the predicted values 6.

At the first assessment, the patients were asked about previous pregnancies and tobacco use during pregnancy according to the questionnaire in the guideline cited above.

### Statistical analysis

For the characterization and summary of the study variables, the mean and standard deviation and median, minimum and maximum values were used for quantitative variables, and absolute and relative frequency were used for qualitative variables.

A paired t-test was used for comparison of spirometric variables in the first and third trimesters. The t-test was applied to compare the groups of nulliparous and multiparous women in relation to spirometric variables in each trimesters, and the Mann-Whitney-test was used for comparing smokers and nonsmokers.

## RESULTS

The initial sample included 220 patients. In total, 11.4% of the patients were excluded due to abortion, 12.7% were excluded due to loss to follow-up, and 22% were excluded due to unsatisfactory spirometric test results. The final sample consisted of 120 pregnant women with 240 valid spirometric exams. The maternal characteristics are presented in Table 1. We analyzed the mean BMI in the first and third trimesters in the two groups, with no significant difference found (first trimester: nulliparous=25.65±5.02; multiparous=26.75±5.81 *p*=0.273); (third trimester: nulliparous=28.70±4.73, multiparous=29.55±5.84 *p*=0.390). There also was no significant difference in the percentage of obese patients in the two groups studied (nulliparous=11/54 (20.4%), multiparous=13/66 (19.7%) *p*=0.927). Table 2 presents the spirometric values obtained in T1 and T3. The absolute and relative values of FVC and absolute and % predicted values of FEV_1_ were significantly reduced in T3 compared with T1.

In terms of parity, as shown in Table 3, 54 patients (45%) were nulliparous, and the other 66 patients (55%) were multiparous. In T1, the multiparous women (0.816±0.059) had smaller FEV_1_/FVC ratios compared with the nulliparous women (0.863±0.049) (*p*<0.0001) and smaller forced expiratory flow in 25% to 75% (FEF_25-75%_), at *p*=0.005 (multiparous women (3.207±0.911) and nulliparous women (3.657±0.797)). In T3, the multiparous women exhibited reductions in the same parameters compared with the nulliparous women: FEV_1_/FVC (multiparous women (30.827±0.047) and nulliparous women (0.856±0.047)) and FEF_25-75%_ (multiparous women (3.253±0.788) and nulliparous women (3.558±0.727)) (*p*=0.00.1 and *p*=0.031, respectively). Maternal age differed among nulliparous (23.00±5.57) and multiparous (29.17±5.96) women at *p*≤0.001. To eliminate the confounding effect of age on absolute spirometry results, patients were categorized according to age: less or equal to 25 and more than 25 years age (Table 4).

Regarding smoking, as shown in Table 5, 17 of the 120 (14.17%) women smoked one to ten cigarettes a day throughout their pregnancies. Some parameters were significantly higher in nonsmokers compared with smokers.

Smokers had lower absolute values of FVC in T1 (3.48±0.37) when compared to nonsmokers (3.70±0.47), *p*=0.042 lower absolute values of FEV_1_ in T3 (2.78±0.29) when compared to nonsmokers (2.99±0.39), *p*=0.039 lower absolute values of FEF_25-75%_ in T3 (3.00±0.69) when compared to nonsmokers (3.46±0.76), *p*=0.024 and lower values of predicted peak expiratory flow (PEF) in T3 (smokers (71.71±11.39) and nonsmokers (80.36±12.53), *p*=0.021.

## DISCUSSION

In this study, it was possible to evaluate the spirometry results of 120 pregnant women during the first and third trimesters of pregnancy. In addition, the results were compared according to parity and smoking. Spirometry is the most widely used pulmonary function test, although it is estimated that approximately 15% of spirometry tests are inadequate 7,8. Because we required two complete examinations (i.e., in T1 and T3), some patients were excluded (22%). However, in one study of the long-term effect of air pollution on respiratory health in adult Swiss woman 9, a 28% loss to follow-up (related to inadequate spirometry tests) was reported, which was greater than the loss during follow-up in the current study.

All spirometric values in the pregnant subjects were within normal ranges. The FVC and FEV_1_ values decreased significantly in the third trimester. Redivo 10 described no changes in pulmonary function in pregnancy 10; however, Grindheim et al. 7 demonstrated differences, particularly in FVC and PEF 7. The results of recent studies agree with our findings 1,11. These reduced values may be due to decreased negative intrapleural pressure due to an upward tilt of the diaphragm caused by the enlarging uterus. Another explanation is the reduction in the alveolar partial pressure of carbon gas, which is caused by hyperventilation during pregnancy 12.

Our results also revealed differences in the spirometric values between smokers and nonsmokers and nulliparous and multiparous women. Compared with the nulliparous women, the multiparous pregnant women had significantly reduced absolute values of FEV_1_/FVC and FEF_25-75%._ This reduction was maintained among the younger multiparous women (≤25 years age) when compared to the younger nulliparous women, suggesting that the decrease found is due to parity. In relation to multiparous women who are more than 25 years old, only the FEV1/FVC ratio in T1 maintained a significant effect. It is important to consider that for this analysis, there was a significant decrease in the number of patients in each group, which can directly influence the calculation of the *p* value. It can be observed that the average of these spirometric variables for different age groups remained similar to the general analysis.

The % predicted value of FEV1/FVC and FEF_25-75%._, which took into account individual characteristics (sex, age, and body mass index), was not significantly different between the two groups. Grindheim et al. 7 found similar results in their study, which analyzed a cohort of 87 healthy pregnant women. The authors concluded that the predicted FVC values in multiparous pregnant women decreased by 4.4% compared with those in nulliparous women (*p*=0.039). No statistically significant differences were observed in the absolute and predicted PEF values. Although obesity was related to changes in spirometry and also to multiparity, there was no difference between the groups in relation to this maternal characteristic.

The absolute values of FVC, FEF_25-75%,_ and FEV_1_ were significantly decreased in smokers compared with nonsmokers. Vergara et al. 13 studied lung function in 136 girls (45.5%) in relation to smoking (active and passive smokers) and investigated repercussions on spirometric parameters. A significant association was found between smoking and spirometric parameters (ANOVA), which was lower in smokers: FVC (*p*=0.001), FEV1 (*p*=0.0001), FEV1/FVC (*p*=0.004), PEF (*p*=0.0001), and FEF25-75% (*p*=0.0001).

Prasad et al. 14 reported a study involving over 100 female smokers and 100 female non-smokers in the age group of 30-40 years in which three lung function tests, FEVI, FVC and PEFR, were employed, and it was observed that all parameters of the three lung function tests were significantly reduced among the smokers compared to the non-smokers.

One limitation is the fact that smoking research was performed only through a structured questionnaire with the possibility of one response (yes or no). Currently, the use of monoximetry is suggested to more accurately discriminate smokers who, for any reason, will declare themselves to be non-smokers. Nonetheless, we must point out that the great majority of studies in the literature that use spirometry for lung function evaluation do not advocate the performance of monoximetry to exclude smoking. In this study, we concluded that the absolute values of FVC, FEF_25-75%,_ and FEV_1_ were significantly decreased in smokers compared with nonsmokers, with significant reductions in absolute and in % predicted FVC and FEV1 values during pregnancy (T1 to T3). Parity and smoking significantly influenced spirometric parameters, and these are important factors that must be considered when analyzing spirometry results during pregnancy.

FVC and FEV_1_ values decreased significantly in the third trimester. The reductions in these parameters may be explained by the upward tilt of the diaphragm caused by the enlarging uterus, causing a decreased negative intrapleural pressure, and because of a reduction in the alveolar partial pressure of carbon gas, which is caused by hyperventilation during pregnancy. Decreased lung function at both the beginning and end of the gestational period is more evident in multiparous women and those who smoke.

## AUTHOR CONTRIBUTIONS

Pastro LD performed spirometry and data analyses and was responsible for the final preparation of the manuscript. Lemos M assisted in the design of the study and spirometry. Fernandes FL provided training regarding spirometry and analyzed spirometric tests. Saldiva SR assisted in the preparation of the project and the recruitment of pregnant women. Vieira SE assisted in the preparation of the project and the recruitment of pregnant women. Romanholo BM performed spirometry. Saldiva PH assisted in the preparation of the project and the final data analysis. Francisco RP assisted in the preparation of the project, manuscript writing and final data analysis.

## Figures and Tables

**Table 1:** Maternal characteristics.

	**Mean±SD**	
**Age (years)**	26.39±6.54	
**Height (meters)**	1.60±0.06	
**Weight (kg)**	T1= 68.55±13.86	
	T3 = 75.97±12.86	
**Gestational age (weeks)**	T1 = 10.60±1.80	
	T3 = 31.70±0.80	
**Ethnicity**	White	38 (31.17%)
	Brown	66 (55.00%)
	Black	16 (13.33%)
**Marital status**	Single	39 (32.50%)
	Married	44 (36.67%)
	Cohabitating	35 (29.17%)
	Separated	2 (1.67%)
**Education level**	Incomplete primary education	30 (25.00%)
	Complete primary education	9 (7.5%)
	Incomplete secondary education	21 (17.50%)
	High school	47 (39.17%)
	Incomplete higher education	4 (3.33%)
	University graduates	9 (7.50%)
**Occupation**	Housewife	35 (29.2%)
	Housemaid	9 (7.5%)
	General Services Assistant	7 (5.8%)
	Hairdresser/Manicurist	5 (4.2%)
	Unemployed	8 (6.7%)
	Other	56 (46.6%)
**Monthly income (Minimum wage (MW): $ 34,700)**	Less than half MW	1 (0.80%)
	Half to equal to one MW	13 (10.80%)
	Between one and three times MW	68 (56.70%)
	Between three and five times MW	15 (12.50%)
	Could not inform	6 (5.00%)
	Would not inform	17 (14.20%)
**Exclusions**	Abortion	25 (11.14%)
	Discontinued participation	18 (8.20%)
	Change of address	10 (4.50%)
	Inappropriate spirometry test	48 (21.80%)

**Table 2:** Comparison of spirometric variables between the first and third trimesters of pregnancy.

Variable		T1	T3	Sig (*p*)
	Mean±SD	Mean±SD		
**Absolute values**	**FVC**	3.670±0.467	3.532±0.459	<0.001
	**FEV_1_**	3.070±0.397	2.964±0.387	<0.001
	**FEV_1_/FVC**	0.837±0.059	0.840±0.049	0.486
	**FEF_25-75%_**	3.409±0.887	3.390±0.773	0.667
	**PEF**	6.179±1.113	6.042±0.964	0.044
**% predicted values**	**FVC**	101.31±10.352	97.20±10.694	<0.001
	**FEV_1_**	99.43±10.880	95.90±11.040	<0.001
	**FEV_1_/FVC**	97.02±6.563	97.53±6.075	0.212
	**FEF_25-75%_**	96.00±24.961	95.80±23.624	0.881
	**PEF**	80.63±14.918	79.23±12.800	0.142

paired t-test.

**Table 3:** Comparison of spirometric variables in the first and third trimesters between nulliparous and multiparous women.

Variable		Nulliparous (n=54)		Multiparous (n=66)		Sig (*p*)
	T	Mean±SD	Median (Min - Max)	Mean±SD	Median (Min - Max)	
**Absolute**	**FVC**	1	3.645±0.537	3.67 (2.65 - 4.89)	3.688±0.400	3.70 (2.89 - 4.70)	0.646
		3	3.531 ±0.509	3.57 (2.67 - 5.00)	3.533 ±0.416	3.49 (2.72 - 4.89)	0.979
	**FEV_1_**	1	3.142±0.419	3.18 (2.29 - 4.07)	3.010±0.317	2.97 (2.15 - 4.12)	0.071
		3	3.015 ±0.407	3.01 (2.24 - 4.00)	2.923 ±0.367	2.91 (2.12 - 4.05)	0.196
	**FEV_1_/FVC**	1	0.863±0.049	0.87 (0.75 - 0.96)	0.816±0.059	0.82 (0.70 - 0.97)	<0.0001
		3	0.856 ±0.047	0.85 (0.75 - 0.98)	0.827 ±0.047	0.84 (0.72 - 0.96)	0.001
	**FEF_25-75%_**	1	3.657±0.797	3.71 (2.10 - 5.29)	3.207±0.911	3.24 (1.63 - 5.79)	0.005
		3	3.558 ±0.727	3.59 (1.96 - 5.33)	3.253 ±0.788	3.26 (1.54 - 4.84)	0.031
	**PEF**	1	6.346±1.176	6.17 (3.87 - 9.40)	6.043±1.049	6.07 (3.78 - 9.76)	0.140
		3	6.137 ±1.032	6.30 (3.77 - 8.84)	5.965 ±0.905	6.12 (3.72 - 9.06)	0.330
**% predicted values**	**FVC**	1	101.48±11.881	101.00 (79 - 126.00)	101.17±9.002	101.50 (83.00 - 123.00)	0.873
		3	98.00 ±12.349	97 (74.00 - 128.00)	96.55 ±9.169	98.00 (75.00 - 121.00)	0.474
	**FEV1**	1	101.57±11.661	102.00 (80.00 - 130.00)	97.68±9.945	98.50 (78.00 - 121.00)	0.051
		3	97.43 ±11.721	97.00 (75.00 - 125.00)	94.65 ±10.374	95.00 (73.00 - 122.00)	0.172
	**FEV_1_/FVC**	1	98.22±5.784	99.00 (84.00 - 108.00)	96.03±7.027	96.00 (83.00 - 116.00)	0.069
		3	97.52 ±6.433	99.00 (83.00 - 107.00)	97.55 ±5.816	98.50 (85.00 - 114.00)	0.981
	**FEF_25-75%_**	1	99.46±22.094	98.00 (51.00 - 161.00)	93.17±26.918	90.00 (53.00 - 173.00)	0.170
		3	96.59 ±21.913	98.50 (46.00 - 151.00)	95.15 ±25.085	94.00 (50.00 - 150.00)	0.741
	**PEF**	1	83.22±14.392	82.00 (50.00 - 114.00)	78.90±15.014	80.00 (49.00 - 127.00)	0.152
		3	80.61 ±11.794	81.00 (51.00 - 108.00)	78.21 ±13.263	79.00 (48.00 - 117.00)	0.352

**Table 4:** Comparison of spirometric variables in the first and third trimesters between nulliparous and multiparous women at ≤ 25 and > 25 years old.

Variable		T	Women ≤25 years		sig (*p)*	Women >25 years		sig (*p*)
	Nulliparous (n=40)		Multiparous (n=20)	Nulliparous (n=14)		Multiparous (n=46)		
	Mean±SD		Mean±SD	Mean±SD		Mean±SD		
**Absolute**	**FVC**	1	3.626±0.532	3.670±0.315	0.691	3.712±0.569	3.696±0.436	0.910
		3	3.515±0.493	3.516±0.318	0.993	3.579±0.573	3.542±0.456	0.801
	**FEV_1_**	1	3.143±0.419	3.038±0.265	0.310	3.141±0.438	2.999±0.412	0.269
		3	3.016±0.411	2.911±0.269	0.302	3.014±0.412	2.928±0.409	0.499
	**FEV_1_/FVC**	1	0.870±0.053	0.829±0.054	0.007	0.847±0.032	0.812±0.062	0.043
		3	0.860±0.050	0.830±0.045	0.026	0.845±0.036	0.826±0.049	0.185
	**FEF_25-75%_**	1	3.721±0.825	3.230±0.673	0.025	3.474±0.707	3.197±1.004	0.342
		3	3.564±0.755	3.220±0.650	0.088	3.542±0.667	3.268±0.849	0.273
	**PEF**	1	6.292±0.987	6.169±1.125	0.666	6.502±1.639	5.989±1.023	0.284
		3	6.121±1.088	5.762±0.911	0.210	6.185±0.892	6.052±0.899	0.630
**% predicted values**	**FVC**	1	101.250±10.970	99.800±7.031	0.538	101.143±14.623	101.761±9.746	0.928
		3	97.450±11.031	94.450±7.577	0.223	99.571±15.907	97.457±9.715	0.644
	**FEV01**	1	101.050±10.651	96.050±7.715	0.065	103.071±14.715	98.391±10.771	0.198
		3	96.625±10.860	91.300±7.349	0.053	99.571±14.095	96.109±11.202	0.325
	**FEV_1_/FVC**	1	97.375±6.270	94.350±5.613	0.074	100.643±3.153	96.761±7.499	0.007
		3	96.475±6.752	95.300±5.516	0.504	100.500±11.202	98.522±5.726	0.239
	**FEF_25-75%_**	1	96.950±22.368	83.150±18.082	0.020	106.643±20.353	97.522±29.061	0.279
		3	92.450±20.551	82.750±17.235	0.075	108.429±22.065	100.543±26.181	0.312
	**PEF**	1	81.929±11.857	75.867±14.202	0.144	86.000±19.022	79.911±15.294	0.273
		3	80.111±13.195	71.063±11.710	0.029	81.571±8.847	80.756±12.958	0.827

**Table 5:** Comparison of spirometric variables in the first and third trimesters between pregnant smokers and nonsmokers.

Variable		Nonsmokers (n=103)		Smokers (n=17)		Sig (*p*)
	T	Mean±SD	Median (Min - Max)	Mean±SD	Median (Min - Max)	
**Absolute**	**FVC**	1	3.70±0.47	3.71 (2.71 - 4.89)	3.48±0.37	3.45 (2.65 - 4.39)	0.042
		3	3.56±0.47	3.53 (2.67 - 5.00)	3.36±0.29	3.32 (2.92 - 3.94)	0.074
	**FEV_1_**	1	3.10±0.40	3.11 (2.15 - 4.12)	2.91±0.32	2.96 (2.29 - 3.38)	0.115
		3	2.99±0.39	2.95 (2.12 - 4.05)	2.78±0.29	2.84 (2.12 - 4.05)	0.039
	**FEV_1_/FVC**	1	0.84±0.06	0.84 (0.70 - 0.97)	0.84±0.05	0.84 (0.71 - 0.96)	0.842
		3	0.84±0.04	0.84 (0.73 - 0.98)	0.83±0.05	0.83 (0.72 - 0.95)	0.154
	**FEF_25-75%_**	1	3.44±0.90	3.43 (1.63 - 5.59)	3.22±0.75	3.26 (1.63 - 4.21)	0.447
		3	3.46±0.76	3.42 (1.55 - 5.33)	3.00±0.69	3.07 (1.54 - 4.09)	0.024
	**PEF**	1	6.25±1.14	6.22 (3.78 - 9.76)	5.73±0.81	5.92 (4.02 - 7.24)	0.055
		3	6.11±0.96	6.20 (3.72 - 9.06)	5.65±0.85	5.58 (3.89 - 6.83)	0.078
**% predicted values**	**FVC**	1	101.95±10.67	102 (79 - 126)	97.41±7.15	96 (87 - 110)	0.064
		3	97.78±11.00	98 (78 - 128)	93.71±7.92	94 (78 - 111)	0.131
	**FEV_1_**	1	100.07±11.11	100 (78 - 130)	95.59±7.63	99 (80 - 108)	0.144
		3	96.70±11.10	96 (73 - 125)	91.06±9.53	95 (75 - 106)	0.081
	**FEV_1_/FVC**	1	97.02±6.62	97 (83 - 116)	97.00±6.39	99 (85 - 105)	0.734
		3	97.79±6.09	99 (83 - 114)	96.00±5.88	97 (85 - 103)	0.252
	**FEF_25-75%_**	1	96.86±25.15	94 (51 - 173)	90.76±23.79	93 (53 - 156)	0.416
		3	97.43±23.54	98 (50 - 151)	85.94±22.30	93 (46 - 135)	0.076
	**PEF**	1	81.63±15.02	82 (49 - 127)	84.57±12.47	78 (50 - 97)	0.092
		3	80.36±12.53	80 (48 - 117)	71.71±11.39	70 (51 - 88)	0.021

Mann-Whitney U-test.
